# Letter from Dr. G. F. J. Colburn

**Published:** 1848-01

**Authors:** G. F. J. Colburn

**Affiliations:** Morristown, N. J.


### ARTICLE V.

### *Letter from* Dr. G. F. J. Colburn *of Morristown, N. J.*

Messrs. Editors:

Knowing that the pages of your valuable Journal are ever open to communications tending to promote the welfare of the profession, and for the suppression of quackery in its ranks, I beg leave to call the attention of its readers to the practice of some dentists, of filling teeth with gold and tin-foil, say three-quarters tin to one of gold, the gold being adroitly wrapped around the the tin, so as to present a surface for the purpose of deceiving the patient, making him pay for gold, when it will in time prove worse than nothing.

Within the last few years I have detected so many cases of this species of quackery, emanating in two instances from dentists of high pretensions, that I deem it but justice to the profession to make them acquainted with the fact and consequences. It is evident that two metals, in connection, are readily acted upon by the sceptic acid of the mouth, causing more or less galvanic action, resulting not only in the destruction of the filling, but frequently of the tooth itself; likewise by vitiating the secretions of the mouth, it causes more or less injury to the surrounding parts, while, at the same time, it creates a bad taste and sensation in the mouth.

From the numerous cases that have come under my observation, I will cite one by way of illustration.

Mr. A., a gentleman residing about eight miles from the village in which I am practising, called upon me for the purpose of ascertaining if any thing could be done to remedy a bad taste, and continual flow of saliva in his mouth, which he suspected proceedcd from a tooth which had been filled nearly a year before. Upon examination, I found he referred to the first molar, on the left side of the upper jaw, which had a large cavity upon its grinding surface, in which I found the remains of a filling composed of tin-foil, much oxydized, and a small portion of gold beneath; the gums around the tooth were of a dark color, and quite sensitive to the touch. Although free from tartar, the secretions of his mouth were extremely vitiated.

Mr. A. appeared surprised when I showed him the filling, and told him it was tin and gold, remarking that he paid for gold, and supposed it was, and that he never, knowingly, had a tin filling in his mouth.

Upon examining the cavity, it appeared black as if charred by fire. After removing from it all impurities, I filled it with cotton saturated with spts. of camphor, at the same time scarifying the gums, requesting him if he did not experience a continuance of his complaint to call in a few days and I would fill it with gold, which he accordingly did. Six months afterwards he told me he had had no trouble from the tooth, and that all unpleasant feelings had left his mouth.

In all the cases that have come under my notice, the results appeared to be uniformly the same, namely, the destruction of the filling, and injury to the teeth and gums.

This practice is decidedly pernicious, and cannot be too strongly deprecated.

What should be thought of him, who, in this age of dental science and investigation, gravely practices such abominable deception upon his patients?

How are we to classify these individuals, shall we call them dentists? or, rather quacks and impostors? The teeth are of too much importance to our whole physical organization to be thus tampered with. All operations upon these organs should be performed not only with the utmost discretion and judgment, but in good faith. At any rate it becomes every honorable practitioner to discountenance and cry out against such mal practice.

G. F. J. COLBURN.

